# The effect of public reporting of acute myocardial infarction on the choice of hospital

**DOI:** 10.1371/journal.pone.0323780

**Published:** 2025-05-27

**Authors:** Mira Kim, Kyungshin Lee, Kyunghee Chae, Chai-Young Jung, Sangmin Lee, Hude Quan, Sukil Kim

**Affiliations:** 1 Department of Preventive Medicine, College of Medicine, The Catholic University of Korea, Seoul, Republic of Korea; 2 Biomedical Research Institute, Inha University Hospital, Seoul, Republic of Korea; 3 Cumming School of Medicine, University of Calgary, Calgary, Canada; 4 Department of Community Health Sciences, University of Calgary, Calgary, Canada; Lithuanian University of Health Sciences, LITHUANIA

## Abstract

This study is to investigate the effect of public reporting of acute myocardial infarction (AMI) care on the people’s choice of hospitals. A cross-sectional study was conducted using an online questionnaire. The survey questions include the awareness and usage of public reporting, and the impact of the public reporting on the choice of hospitals. The difference in responses before and after acquiring information about public reporting was compared using multinomial logistic regression. Following a thorough validity check, 740 respondents are included in the final survey data set. The average age of respondents was 38.7 years (SD: 11.8), with 75.3% being female. Age distribution was as follows: 26.3% in their 20s, 23.5% in their 30s, 30.0% in their 40s, and 20.2% in their 50s. Most participants (73.7%) lived in metropolitan areas, and 75.1% had a university degree or higher. Before providing information about public reporting of AMI care, 62.8% of respondents selected ‘nearby hospitals’ as the best option for AMI patients, followed by ‘famous hospitals’, ‘usual hospital’, and ‘hospitals with good rates’. Non-health-related occupation shows significantly changed results of hospital choice between before and after obtaining public reporting information (p < 0.001). Publicly available hospital quality ratings can influence people’s choice of hospital and increase the risk of selecting a hospital with a good rating than the nearest hospital which is recommended for AMI patients. Policy-makers need to stress the importance of choosing the nearest hospital when AMI symptoms occur in addition to hospital ratings in the public reporting.

## Introduction

### Public reporting on Acute Myocardial Infarction (AMI) care in Korea

Public release of hospital quality information, referred to as public reporting, is intended to improve patients’ knowledge about hospital quality and offer potential patients the possibility of making a well-informed choice of their hospitals [1,2]. Patients are provided with comparative information such as indicators for measuring the quality of various care providers or different healthcare facilities. Patients are expected to actively participate in choosing their health care providers so that they could benefit from high quality treatment opportunities.

A preliminary public quality report on AMI care in Korea has shown that the AMI mortality ratio has regional variations indicating differences in the quality of care and the structure among medical care benefit agencies and in the assessment of the treatment process [3]. A regional comparison of age-standardized mortality rates for heart disease showed that the area with the highest rate (36.5) had a mortality rate 1.93 times greater than the area with the lowest rate (18.9). Mortality rates in the southeastern region were notably higher than in other areas, including Seoul, the capital of South Korea [4].

Therefore, the Health Insurance Review and Assessment Service (HIRA) has assessed the quality of AMI treatment annually from 2008 to 2013 to improve the quality of AMI care by providing results of AMI care process to the public. Evaluation of hospital care quality for AMI are divided into five grades according to their scores, with smaller scores indicating better hospitals for AMI treatment. The evaluation results have been publicly disclosed on the Korea Healthcare Quality Assessment website to help people in making informed hospital choices. [5].

### Choice of hospitals

With over 90% of privatized hospitals in Korea, choosing a hospital to receive medical care for Koreans can be a complicated and difficult process. Choosing unsuitable facilities may result in delays of treatments, which may increase patient’s dissatisfaction with their health care service and lead to undesirable clinical outcomes [6,7]. The choice of hospital for patients is influenced by various factors such as previous experiences or opinions of their acquaintances [8–10], reputations of the hospitals, and recommendations from their general physicians (GPs) [11,12]. Several studies have revealed that hospital quality information can have a significant positive impact on patients’ choices [13–15]. However, it may mislead patients who are sensitive to differences in hospital quality measured by public quality ratings to ‘higher-quality’ hospitals [16].

The number of beds in Korean public hospitals is far lower than the Organization for Economic Cooperation and Development (OECD) average (1.2 vs. 2.8 beds per every 1,000 people) [17,18]. However, these cases could be successfully managed at physicians’ clinics in the community. Competition in the healthcare market and shortcomings in the care delivery system have resulted in tertiary-care hospitals covering a larger share of ambulatory care services compared to community hospitals.

Tertiary-care hospitals usually located in the metropolitan area are superior to other general hospitals in terms of medical personnel, facilities, and equipment [19]. This may potentially be due to the fact that medical expenses are concentrated in tertiary-care hospitals located in Seoul, Korea [20].

The Korean healthcare system has a unique feature that allows patients to freely access any medical institution, regardless of their location or type [21]. This often encourages patients in Korea to choose tertiary-care hospitals over smaller local clinics. While this freedom of choice may benefit patients, it can also create challenges, such as an over-concentration of medical resources and excessive visits to certain facilities. For instance, as more patient flock to tertiary-care hospitals in Seoul, smaller local clinics and public hospitals receive fewer patients, leading to an imbalance in the distribution of medical services. The high concentration of patients at prestigious tertiary hospitals in Seoul remains a concern. Although co-payments increase with each hospital visit to help reduce patient numbers at these institutions, this measure has not effectively discouraged patients from frequenting these hospitals [22].

### Purpose of this study

The purpose of this study was to observe the effect of public reporting of AMI on people’s choice of hospitals when there is no restriction in choosing a hospital. The effect of public reporting will be evaluated by analyzing any differences in respondents’ hospital choices before and after obtaining information on hospital rating (public reporting). Effects were compared between individuals with health-related and those with non-health-related occupations. Factors affecting the choice of hospitals for AMI and cancer treatment were also compared to determine whether there were differences in choices of hospitals for receiving medical care for acute or chronic diseases.

## Materials and methods

### Participant recruitment

Participants for this study were recruited from Dec. 9, 2020 to Jan. 3, 2021 using a snowball sampling, also known as chain sampling. In this type of sampling, initial participants are recruited in a non-random manner and are then asked to refer other individuals who may fit the study’s selection criteria. We first identified six individuals initially from authors of this study, consisting of three health-related and three non-health-related workers in their 20s, 30s, or 40s. These six individuals along with other respondents of the survey were asked to share the survey link with local friends and relatives. Each respondent was paid a $2 e-gift card for their participation.

We defined ‘Health-related workers’ as individuals who work in the healthcare and medical industry (e.g., hospitals, public health centers, pharmacies) or in related research institutions, as well as those whose family members have health-related occupations. In contrast, the ‘Non-health-related workers’ was defined as individuals working in various industries and service sectors outside of healthcare institutions, categorized based on responses to question 20 on the occupation section of the survey. Given that the ‘Health-related workers’ group possesses substantial medical knowledge and a greater understanding of healthcare processes, the study aims to analyze the impact of public reports on hospital selection among patients by comparing this group with the ‘Non-health-related workers’ group, who typically have less medical knowledge.

### Online survey

A cross-sectional study was conducted using an online questionnaire. The survey questions were reviewed by two epidemiologists and two cardiologists. A short background and the objective of the study were presented, followed by several questions to investigate the awareness and usage of public reporting and the impact of the public reporting on the choice of hospitals.

The first and second sections of the questionnaire aim to assess the impact of HIRA’s public reporting on hospital selection for AMI and cancer treatment. It included questions about hospital choice before and after acquiring information about public reporting. After two brief explanations (covering information from HIRA hospital quality reports and the importance of timely treatment for acute myocardial infarction), participants were asked again to choose a hospital, and their choices before and after the interventions were then statistically compared. As a supplementary third micro-intervention, a brief explanation of the need for treatment in the case of a cancer was provided. Participants were then asked again about the procedure for choosing a hospital, allowing for a comparison between hospital selection in acute and non-acute hospital admission situations. The analysis distinguished between individuals with and without reference to health care professions.

The third section consists of questions aimed at assessing the respondent’s level of knowledge regarding AMI (such as symptoms, necessary information, sources of information, etc.). The final section captured demographic characteristics such as gender, age, marital status, education, residence, income, and occupation. The survey included a Likert scale, score (4-point scale), multiple choice, short-answer, and open-ended questions. The survey took less than 10 minutes to complete.

### Data collection

We developed a website for individuals to fill out the questionnaire via a computer or mobile phone. The URL was distributed to individuals using a free mobile instant messaging application with free text and free call feature (KakaoTalk©) for both Android and iOS users. An example of how the survey shows on a mobile phone is presented in S1 Fig.

### Statistical analysis

Demographic characteristics, general knowledge about AMI, hospital choice for AMI or cancer treatment, and factors affecting hospital choice were compared between participants with a health-related occupation and those with a non-health-related occupation using t-test for continuous variables and χ² test for categorical variables. All statistics were presented with frequency (%) for categorical variables and mean (SD) for continuous variables. Continuous variables were compared using Mann-Whitney U test and categorical variables were compared using Chi-square test and Fisher’s exact test.

Respondents with family members having health-related occupations were grouped into the health-related occupation group because these respondents would have more knowledge about the disease or choice of hospital by their associations with such family members than respondents who did not have a family member with health-related occupations.

This study used McNemar’s test, a response analysis to paired categorical data performed twice before and after training on one subject. Furthermore, for continuous variables, the same individual was repeatedly measured to evaluate selection changes over time before and after acquiring additional information for the choice of hospital, and statistical significance was performed with Friedman’s test considering that the hospital selection score was a ranking measure. All data analyses were performed using SAS Enterprise Guide 7.12. Statistical significance for all analyses was set *a priori* at α < 0.05.

### Ethical statement

Completion of the survey was voluntary. We provided a statement that by filling out and returning the survey, the participants give their informed consent. Before starting the survey, participants were provided with a brief introduction explaining that the purpose of the survey is to study the factors influencing hospital choice among patients with acute and chronic illnesses. The introduction also clarified that responses would remain anonymous and be used solely for research purposes. Participants were then asked to confirm their willingness to proceed by checking a box.

All responses were treated anonymously and confidentially. Ethics approval for this study was obtained from the Institutional Review Board of Catholic University of Korea (MC20QISI0154).

## Results

### Study population

Of 740 individuals who participated in the survey, 494 (66.7%) completed their questionnaires while 241 individuals did not complete their questionnaires and five individuals had errors in their birth dates.

Table 1 shows general characteristics of the overall respondents as well as those with health-related or non-health-related occupation. The mean age of respondents was 38.7 (SD:11.8) years and 75.3% were females. Percentage of respondents according to age group was 26.3% for those in their 20s, 23.5% for those in their 30s, 30.0% for those in their 40s, and 20.2% for those in their 50s. Participants living in the metropolitan area accounted for 73.7% of the respondents and 75.1% of all respondents had a university education or higher. There were 163 (33%) respondents who were in the group with health-related occupation, of which 52.1% were nurses, 14.7% were hospital staffs, and 11.0% were medical doctors. There were significant differences in the mean age, age group, and gender between those with a health-related occupation and those with a non-health-related occupation. Those with a health-related occupation were younger than those with a non-health-related occupation (35.8 years vs. 40.2 years, *p* < 0.001). Female proportion in those with a health-related occupation was greater (83.4% vs. 71.3%, *p* = 0.003). The proportion of those with a health-related occupation living in the metropolitan area was greater (81.0% vs. 70.1%, *p* = 0.010). The proportion of those with a health-related occupation having a university education or higher was also higher (27.6% vs. 13.9%, *p* < 0.001).

**Table 1:** General characteristics of the respondents.

		(N, %)		
Characteristics	Total (n = 494)	Health related occupation (including family member)		
		Yes (n = 163)	No (n = 331)	*p value*
	494 (100)	163 (33.0)	331 (67.0)	
**Age, mean (SD)**	38.7 (11.8)	35.8 (12.3)	40.2 (11.4)	<0.001
**Age group**				
20 - 29	130 (26.3)	64 (39.3)	66 (19.9)	<0.001
30 - 39	116 (23.5)	34 (20.9)	82 (24.8)	
40 - 49	148 (30.0)	36 (22.1)	112 (33.8)	
> 50	100 (20.2)	29 (17.8)	71 (21.5)	
**Gender**				
Male	122 (24.7)	27 (16.6)	95 (28.7)	0.003
Female	372 (75.3)	136 (83.4)	236 (71.3)	
**Residency**				
Metropolitan area	364 (73.7)	132 (81.0)	232 (70.1)	0.010
Other than metropolitan area	130 (26.3)	31 (19.0)	99 (29.9)	
**Education level**				
High School or lower	123 (24.9)	43 (26.4)	80 (24.2)	<0.001
Undergraduate	280 (56.7)	75 (46.0)	205 (61.9)	
Graduate school or higher	91 (18.4)	45 (27.6)	46 (13.9)	

### General knowledge about AMI

Table 2 shows respondents’ general knowledge about AMI. Respondents who selected little or not at all when asked about awareness of their symptoms, coping, and treatment methods for AMI accounted for 49.4% of all subjects. This percentage differed between those having a health-related occupation and those having a non-health-related occupation (24.6% vs. 61.6%, *p* < 0.001). Majority of respondents obtained their information about AMI through ‘Mass media, such as TV’ (40.6%) or ‘Internet or books’ (34.8%). Those with a non-health-related occupation were more likely to obtain their source of information about AMI through ‘Mass media, such as TV’ (51.1% vs. 20.2%, *p* < 0.001) but less likely through health professionals (6.6% vs. 31.3%, *p* < 0.001) than those with a health-related occupation. Regarding their responses to the most useful information to cope with AMI, ‘early symptoms and self-diagnosis’ had the highest proportion (75.1%), followed by ‘preventive measures’ (38.9%), ‘hospital information for AMI treatment’ (30.6%), and ‘hospital ratings based on the public reporting of AMI care quality’ (9.9%). People with a non-health-related occupation considered ‘early symptoms and self-diagnosis of AMI’ less useful (72.2% vs. 81.0%, *p* = 0.034) while they considered ‘hospital ratings for AMI care quality’ to be the most useful information for coping with AMI (12.7% vs. 4.3%, *p* = 0.003).

**Table 2:** General knowledge about AMI.

		(N, %)		
Questions	Total (n = 494)	Health related occupation		
		Yes (n = 163)	No (n = 331)	*p value*
**1. Awareness of the symptoms, coping and treatment methods for AMI**				
Very well	48 (9.7)	32 (19.6)	16 (4.8)	<0.001
Some	202 (40.9)	91 (55.8)	111 (33.5)	
Little	190 (38.5)	36 (22.1)	154 (46.5)	
Not at all	54 (10.9)	4 (2.5)	50 (15.1)	
**2. Information resource of AMI**				
Health professionals	73 (14.8)	51 (31.3)	22 (6.6)	<0.001
Acquaintances	133 (10.7)	28 (10.5)	105 (10.7)	
Mass media such as TV	619 (49.6)	96 (36.0)	523 (53.4)	
Internet or books	172 (34.8)	64 (39.3)	108 (32.6)	
Other	6 (1.2)	4 (2.5)	2 (0.6)	
**3. Most useful information to cope with AMI (multi-selectable)**				
Preventive measure of AMI	192 (38.9)	64 (39.3)	128 (38.7)	0.899
Early symptoms and self-diagnosis of AMI	371 (75.1)	132 (81.0)	239 (72.2)	0.034
Hospital information for AMI treatment	151 (30.6)	46 (28.2)	105 (31.7)	0.427
Hospital ratings for AMI care quality	49 (9.9)	7 (4.3)	42 (12.7)	0.003
Other	5 (1.0)	1 (0.6)	4 (1.2)	0.534

### Public reporting and hospital choice

Table 3 shows hospital choice before and after acquiring information on public reporting and clinical information of AMI. There were 37.4% of respondents who selected ‘little’ and 23.7% who selected ‘not at all’ when asked about their awareness of the HIRA public reporting. This differed by having a health-related or a non-health-related occupation (little: 34.4% vs. 39.0%; Not at all: 13.5% vs. 28.7%, both *p* < 0.001). Before providing information about public reporting and clinical information about AMI, 62.8% of respondents selected ‘nearby hospitals (close)’ as the best option for AMI patients, followed by ‘famous hospitals recommended by acquaintances (famous)’ (14.4%), ‘usual hospital (favorite)’ (10.5%), and ‘hospitals that received good rate for AMI treatment by the HIRA (good rate)’ (9.9%). There was no significant difference in hospital choice between those with a health-related occupation and those with a non-health-related occupation.

**Table 3:** Hospital choice for AMI treatment.

		(N, %)		
Questions	Total (n = 494)	Health related occupation		
		Yes (n = 163)	No (n = 331)	*p value*
**1. Awareness of Public reporting of the HIRA**				
Very well	35 (7.1)	16 (9.8)	19 (5.7)	<0.001
Some	157 (31.8)	69 (42.3)	88 (26.6)	
Little	185 (37.4)	56 (34.4)	129 (39.0)	
Not at all	117 (23.7)	22 (13.5)	95 (28.7)	
**2. Which of the following hospital will you visit if you or your family have AMI?**				
Favorite	52 (10.5)	16 (9.8)	36 (10.9)	0.238
Close	310 (62.8)	102 (62.6)	208 (62.8)	
Famous	71 (14.4)	29 (17.8)	42 (12.7)	
Good rate	49 (9.9)	15 (9.2)	34 (10.3)	
No idea	12 (2.4)	1 (0.6)	11 (3.3)	
Public reporting on AMI care*: Since AMI requires immediate treatment, hospital care quality of AMI treatment is evaluated whether the treatment (oral medication, stents, etc.) was done in a reasonable time. The results of the public reporting are divided into five grades according to the score. Smaller numbers indicate better hospitals for the AMI treatment. **3. To what extent would you consider hospital rating for your future hospital choice for AMI treatment?**				
Considerably	246 (49.8)	75 (46.0)	171 (51.7)	0.497
Some	208 (42.1)	74 (45.4)	134 (40.5)	
Not at all	40 (8.1)	14 (8.6)	26 (7.9)	
Clinical Information about AMI**: AMI is a life-threatening condition that occurs when blood flow decreases or stops to a part of the heart, causing damage to the heart muscle. The most common symptom is chest pain or discomfort which may travel into the shoulder, arm, back, neck, or jaw. AMI is high-mortality disease and requires immediate treatment. **4. Which of the following hospital will you visit if you or your family have AMI?**				
Favorite	32 (6.5)	10 (6.1)	22 (6.6)	0.160
Close	287 (58.1)	101 (62.0)	186 (56.2)	
Famous	66 (13.4)	24 (14.7)	42 (12.7)	
Good rate	100 (20.2)	28 (17.2)	72 (21.8)	
No idea	9 (1.8)	0 (0.0)	9 (2.7)	

After receiving detailed information about hospital ratings for AMI care from HIRA’s public report, the majority of respondents answered they would consider this information for their future hospital choice to some extent (42.1%) or considerably (49.8%). This did not differ by whether they had a health occupation or not (*p* = 0.497). After acquiring information about the public reporting of the HIRA, 11.5% of those with a non-health-related occupation and 8% of those with a health-related occupation changed their original responses to ‘good rate’. Difference between the two groups was not statistically significant (*p* = 0.160) (Table 3).

Table 4 shows significantly changed results of hospital choice between before and after obtaining public reporting information (*p* < 0.001). Compared to the health-related occupation group, there was difference in pre_response of hospital choice among respondents whose post response was changed by non-health-related occupation group (non-health related occupation group: p < 0.001 vs health related occupation group: p = 0.095). Respondents who did not change their pre-response of hospital choice to ‘good rate’ accounted for 73.5% in the non-health-related occupation group and 80.0% in the health-related occupation group. Among the respondents who changed the post-response of hospital choice to ‘good rate’, 14.4% of non-health-related occupations and 7.8% of health-related occupations answered their pre-response of hospital choice of ‘close’.

**Table 4:** Change of hospital choice between pre-response and post-response.

	(N, %)													
Pre-response^*^	Post-response^**^													
	Total (*p < 0.001*)				Health related occupation									
					Yes (*p = 0.095*)					No (*p < 0.001*)				
	Favorite	Close	Famous	Good rate	No idea	Favorite	Close	Famous	Good rate	Favorite	Close	Famous	Good rate	No idea
Sum	32	287	66	100	9	10	101	24	28	22	186	42	72	9
Favorite	22 (42.3)	14 (26.9)	7 (13.5)	9 (17.3)	0 (0.0)	6 (37.5)	4 (25.0)	3 (18.8)	3 (18.8)	16 (44.4)	10 (27.8)	4 (11.1)	6 (16.7)	0 (0.0)
Close	7 (2.3)	241 (77.7)	22 (7.1)	38 (12.3)	2 (0.6)	2 (2.0)	85 (83.3)	7 (6.9)	8 (7.8)	5 (2.4)	156 (75.0)	15 (7.2)	30 (14.4)	2 (1.0)
Famous	2 (2.8)	23 (32.4)	33 (46.5)	13 (18.3)	0 (0.0)	1 (3.4)	10 (34.5)	14 (48.3)	4 (13.8)	1 (2.4)	13 (31.0)	19 (45.2)	9 (21.4)	0 (0.0)
Good rate	1 (2.0)	9 (18.4)	1 (2.0)	37 (75.5)	1 (2.0)	1 (6.7)	2 (13.3)	0 (0.0)	12 (80.0)	0 (0.0)	7 (20.6)	1 (2.9)	25 (73.5)	1 (2.9)
No idea	0 (0.0)	0 (0.0)	3 (25.0)	3 (25.0)	6 (50.0)	0 (0.0)	0 (0.0)	0 (0.0)	1 (100.0)	0 (0.0)	0 (0.0)	3 (27.3)	2 (18.2)	6 (54.5)

*Notes*:

* Responses before acquiring information

** Responses after acquiring information, Excluded ‘No idea’ of post response of health-related occupation

Table 5 shows results of Friedman test regarding the score of hospital choice before and after obtaining public reporting information. Among hospital selection factors that differed between before and after obtaining public reporting information, responses to choosing ‘close’ and ‘good rate’ hospitals were increased, while responses to choosing ‘favorite’ and ‘famous’ hospitals were decreased. Compared to the health-related occupation group, responses for ‘close’ and ‘good rate’ hospitals in the non-health-related occupation group were significantly increased, while those for ‘favorite’ and ‘famous’ hospitals were decreased significantly (favorite, *p* < 0.001; close, *p* = 0.028; famous, *p* < 0.001; and good rate, *p* = 0.004).

**Table 5:** Pre- and post-score of hospital choice by health occupation group.

				(mean, SD)					
Score of hospital choice	Total			Health related occupation					
				Yes			No		
	Pre^*^	Post^**^	p value	Pre	Post	p value	Pre	Post	p value
Favorite	2.10 (1.13)	1.94 (1.06)	<0.001	2.13 (1.14)	1.93 (1.03)	<0.001	2.08 (1.12)	1.94 (1.08)	<0.001
Close	2.77 (1.04)	2.88 (1.03)	0.005	2.80 (1.08)	2.95 (1.06)	0.083	2.75 (1.03)	2.84 (1.01)	0.028
Famous	2.51 (0.97)	2.43 (0.96)	0.010	2.50 (1.01)	2.47 (0.98)	0.896	2.51 (0.95)	2.40 (0.95)	<0.001
Good rate	2.63 (1.20)	2.76 (1.16)	0.001	2.58 (1.13)	2.65 (1.14)	0.131	2.65 (1.24)	2.82 (1.17)	0.004

*Notes*:

* Responses before acquiring information

** Responses after acquiring information

### Differences in hospital choice criteria between AMI and cancer

Table 6 shows hospital choices for cancer treatment. Respondents who selected ‘hospitals recommended by acquaintances (famous)’ as the best option for cancer treatment had the highest percentage (28.9%), followed by those who selected ‘hospitals that received good rates for cancer treatment by the HIRA (good rate)’ (27.7%) and those who selected ‘no idea’ (22.7%). For those with a non-health-related occupation, those who selected ‘good rate’ (31.1%) as the best option for cancer treatment accounted for the most, followed by those who selected ‘famous’ (27.2%) and those who selected ‘no idea’ (19.6%).

**Table 6:** Hospital choice for cancer treatment.

		(N, %)		
Questions		Health related occupation		
	Total	Yes	No	*p value*
**1. Which of the following hospital will you visit if you or your family have cancer?**				
Favorite	19 (3.8)	8 (4.9)	11 (3.3)	0.016
Close	83 (16.8)	21 (12.9)	62 (18.7)	
Famous	143 (28.9)	53 (32.5)	90 (27.2)	
Good rate	137 (27.7)	34 (20.9)	103 (31.1)	
No idea	112 (22.7)	47 (28.8)	65 (19.6)	
**2. To what extent would you consider hospital rating for your future hospital choice for cancer treatment?**				
Considerably	289 (58.5)	87 (53.4)	202 (61.0)	0.141
Some	177 (35.8)	63 (38.7)	114 (34.4)	
Not at all	28 (5.7)	13 (8.0)	15 (4.5)	

For those with a health-related occupation, those who selected ‘famous’ (32.5%) as the best option for cancer treatment accounted for the most, followed by those who selected ‘no idea’ (28.8%) and those who selected ‘good rate’ (20.9%). There was significant difference between two groups in choosing hospital for cancer treatment (*p* = 0. 016). After acquiring information of public reporting on cancer treatment provided by the HIRA, 58.5% of respondents answered ‘considerably’ and 35.8% responded ‘some’ when asked to what extent they would consider the information of public reporting for their future hospital choice for cancer treatment.

The most important factor influencing the choice of hospital for both AMI and cancer treatment was good rate reported by the HIRA (AMI: 57.3%; Cancer: 69.8%). Distance to the hospital with traffic (AMI: 17.2%; Cancer: 3.2%) was the next important factor. Size and facilities of the hospital were also important when choosing a hospital. While there was no significant difference in choosing hospital for AMI treatment (*p* = 0. 162) between the two occupation groups, there was significant difference in selecting hospital for cancer treatment (*p* = 0. 045) between the two occupation groups (Table 7).

**Table 7:** Factors affecting patient’s hospital choice for AMI vs. cancer.

		(N, %)		
Factors affecting hospital choice		Health related occupation		
	Total	Yes	No	*p value*
**AMI**				
Reasonable price	17 (3.4)	3 (1.8)	14 (4.2)	0.162
Hospitals received good rates for cancer or AMI treatment from the HIRA	283 (57.3)	91 (55.8)	192 (58.0)	
Recommendation of acquaintances	26 (5.3)	6 (3.7)	20 (6.0)	
Close distance and convenient transportation	85 (17.2)	29 (17.8)	56 (16.9)	
Patients’ satisfaction with hospital services	21 (4.3)	7 (4.3)	14 (4.2)	
The size of hospitals and high-tech facilities	60 (12.1)	25 (15.3)	35 (10.6)	
Other	2 (0.4)	2 (1.2)	0 (0.0)	
**Cancer**				
Reasonable price	19 (3.8)	5 (3.1)	14 (4.2)	0.045
Hospitals received good rates for cancer or AMI treatment from the HIRA	345 (69.8)	114 (69.9)	231 (69.8)	
Recommendation of acquaintances	35 (7.1)	12 (7.4)	23 (6.9)	
Close distance and convenient transportation	16 (3.2)	2 (1.2)	14 (4.2)	
Patients’ satisfaction with hospital services	19 (3.8)	2 (1.2)	17 (5.1)	
The size of hospitals and high-tech facilities	58 (11.7)	27 (16.6)	31 (9.4)	
Other	2 (0.4)	1 (0.6)	1 (0.3)	

## Discussion

Results of this study highlight that public reporting may mislead the choice of hospital for patients with AMI to select a hospital with a good rating rather than choosing the nearest hospital. Before providing clinical information and public reporting of AMI to respondents, most respondents answered that they would go to the nearest hospital after the onset of AMI symptoms. Despite the fact that most respondents had little knowledge about AMI, the majority of respondents had the nearest hospital as their choice. This is crucial as appropriate timing of treatment for AMI is essential to have better outcomes.

However, after receiving information about hospital ratings for AMI care from HIRA’s public report and clinical information of AMI, the number of respondents who chose hospitals with good rating (1 or 2 grade) showed a significant increase. Although HIRA publicly discloses both hospital ratings and information on available hospitals for AMI care [23], these information did not affect respondent’s choice of hospital because they were not aware of it. This finding is consistent with previous studies.

According to de Groot’s study of new surgical patients in the Netherlands, patients who have compared hospitals more often used public information for their hospital choice than patients who have not compared hospitals (12.7% vs. 1.5%, p < 0.001). Patients who compared hospitals assigned lower importance to hospital distance (p = 0.041) than information on wound infections and patient respect [9]. The results from analyzing data from a survey of 467 patients in Minnesota suggest that the factors considered of most significant importance include ‘reputation of the healthcare organization’ rather than ‘distance’ (79.3 ~ 96.1% vs. 32.6 ~ 55.0) [24]. Meanwhile, in the early days of the publication of Public Performance Reports, a survey of patients who underwent CABG surgery found that 56 of the 474 (12%) said they knew of the Consumer Guide before their operation. Only 11 patients (2%) reported that the information influenced their choice of hospital [25].

Unlike chronic diseases such as cancer, it is of importance to visit the nearest hospital where the appropriate treatment is available as soon as the onset of AMI symptom occurs since early treatment is critical for the prognosis of AMI [26]. According to the report, the average time taken to arrive at tertiary hospitals after the onset of AMI is 30 minutes later than the time taken to arrive at general hospitals in Korea [5].

Prehospital delays for AMI treatment are known to increase the risk of mortality [27,28]. Patients with AMI who tend to choose hospitals based on hospital ratings are often at risk of missing the ‘golden time’ for proper treatment while looking for hospitals with a good rating. In addition, outside the metropolitan area in Korea, 80% of patients with AMI die because of missing the ‘golden time’ for treatment while being transferred to the emergency room after the onset of AMI symptoms [29].

The fact that hospital ratings are considered more when selecting hospitals for cancer treatment than AMI treatment indicates that there are differences in factors to consider when choosing a hospital for acute (AMI) and chronic disease (cancer). When ranking hospitals, it is necessary to interpret the data appropriately. However, the public reporting offers no guidance as to the relative benefit of specific measures. For example, when choosing a hospital for medical care of a patient with pneumonia, the guidance in different factors including appropriate timing for antibiotics (i.e., within four hours) and what correct antibiotics should be given is more important [30]. Although there are few studies evaluating the effect of severity of illness or insurance type on hospital choice [31,32], the evidence for hospital choice according to disease type remains limited.

Despite a small proportion of respondents (9.9%) who considered hospital ratings as the most useful information for AMI treatment, more than half (57.3%) of respondents selected ‘hospitals received good rates for cancer or AMI treatment from the HIRA’ as the most important factor for their future hospital choice for AMI treatment. This can be interpreted in respondents’ belief that the hospital with a good rating for AMI care quality is the best hospital with great doctors’ expertise.

Another interesting finding was the association between education level and the choice of hospital. In our study, additional information about clinical characteristics of the disease or hospital rating had no effect on respondents’ choice of hospital by education level (S2 Table). However, a previous study [33] has shown a significant relationship between education level and the choice of highly specialized hospital. Thus, additional study is needed to investigate various factors that might influence the choice of hospitals.

The strength of this study was that we used mobile devices and social media platforms to collect data for our survey. This way of collecting data is cost-effective as it enables access to large and diverse individuals quickly. It takes less time than traditional methods to obtain data for analysis. In addition, this research is easily replicable using the standardization of data collection process.

This study has some limitations. First, results could not be generalized to the entire Korean population because a snowball sampling method was used to select the study population. Most respondents were young with age less than 40 years. This is because mobile and social media platforms are more popular in this age group [34,35]. In addition, respondents were concentrated in the metropolitan area. The approach of snowball sampling might lead to overrepresentation of younger age groups with health-related occupation rather than major age groups with AMI.

When providing clinical information and public reporting of AMI in the survey to observe changes in hospital choice for AMI treatment between before and after information acquisition, we did not inform them that when someone had the onset of AMI symptom, he or she should go to the nearest hospital as soon as possible because this might lead respondents to choose the ‘close hospitals’ for the question of hospital choice. In addition, the definition of ‘good rate’ was not specifically presented in the survey questions. We assumed that respondents considered the first and second grades out of five as good.

## Conclusions

Our study found that publicly available hospital quality ratings influenced people’s choice of hospital, increasing the chance of selecting a highly rated hospital over the nearest one, which is typically recommended for AMI patients. Thus, policy-makers need to stress the importance of choosing the nearest hospital when AMI symptoms occur in addition to hospital ratings in public reporting.

## Supporting information

S1 Fig Screen shot of survey through Kakaotalk. (TIF)

S2 Table Hospital choice by education level. (DOCX)

S3 File Survey questions. (DOCX)
