# Correction: The effect of public reporting of acute myocardial infarction on the choice of hospital

**DOI:** 10.1371/journal.pone.0333102

**Published:** 2025-09-23

**Authors:** Mira Kim, Kyungshin Lee, Kyunghee Chae, Chai-Young Jung, Sangmin Lee, Hude Quan, Sukil Kim

The authors are listed out of order. Please view the correct author order, affiliations, and citation here:

Kyungshin Lee^1^, Mira Kim^1^, Kyunghee Chae^1^, Chai-Young Jung^2^, Sangmin Lee^3^, Hude Quan^4^, Sukil Kim^1^

**1** Department of Preventive Medicine, College of Medicine, The Catholic University of Korea, Seoul Republic of Korea, **2** Biomedical Research Institute, Inha University Hospital, Seoul, Republic of Korea, **3** Cumming School of Medicine, University of Calgary, Calgary, Canada, **4** Department of Community Health Sciences, University of Calgary, Calgary, Canada

Lee K, Kim M, Chae K, Jung C-Y, Lee S, Quan H, et al. (2025) The effect of public reporting of acute myocardial infarction on the choice of hospital. PLoS One 20(5): e0323780. https://doi.org/10.1371/journal.pone.0323780
