# Supplementary figures and images for: The effect of public reporting of acute myocardial infarction on the choice of hospital
Source: PLoS One. 2025 May 27;20(5):e0323780. doi: 10.1371/journal.pone.0323780 (PMC12111679; doi:10.1371/journal.pone.0323780)

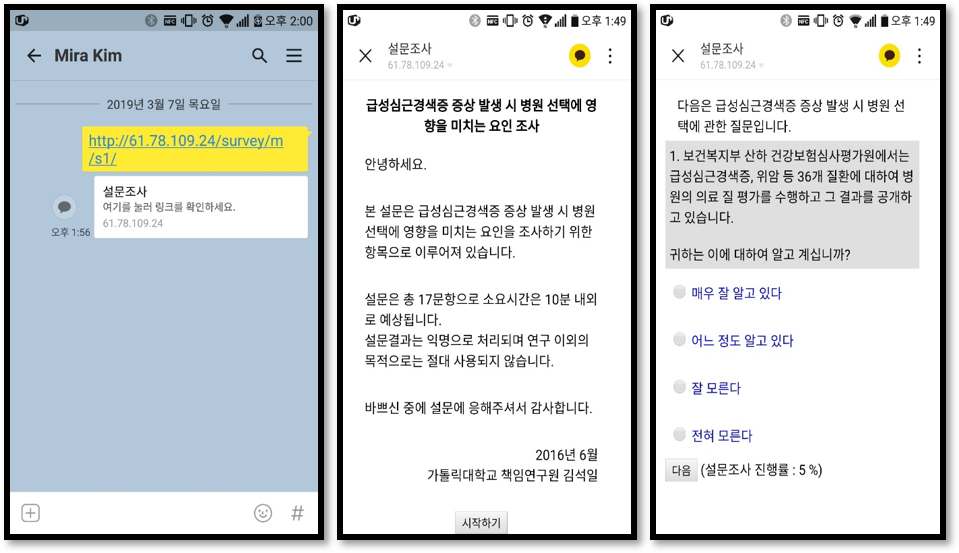

Supplement: S1 Fig — (TIF) [file pone.0323780.s001.tif]
