# Supplementary material for: The effect of public reporting of acute myocardial infarction on the choice of hospital
Source: PLoS One. 2025 May 27;20(5):e0323780. doi: 10.1371/journal.pone.0323780 (PMC12111679; doi:10.1371/journal.pone.0323780)
Supplement: S2 Table — (DOCX) [file pone.0323780.s002.docx]

**S2 Table. Hospital choice by education level**

| **Questions** | **Total (n=494)** | **High School or lower**  **(n=123)** | **Undergraduate (n=280)** | **Graduate school or higher**  **(n=91)** | ***p value*** |
| --- | --- | --- | --- | --- | --- |
| **Which of the following hospital will you visit if you or your family have AMI?** | | | | | |
| Favorite | 32 (6.5) | 11 (8.9) | 17 (6.1) | 4 (4.4) | 0.216 |
| Close | 287 (58.1) | 69 (56.1) | 155 (55.4) | 63 (69.2) |  |
| Famous | 66 (13.4) | 16 (13.0) | 43 (15.4) | 7 (7.7) |  |
| Good rate | 100 (20.2) | 23 (18.7) | 60 (21.4) | 17 (18.7) |  |
| No idea | 9 (1.8) | 4 (3.3) | 5 (1.8) | 0 (0.0) |  |
