# Supplementary material for: The effect of public reporting of acute myocardial infarction on the choice of hospital
Source: PLoS One. 2025 May 27;20(5):e0323780. doi: 10.1371/journal.pone.0323780 (PMC12111679; doi:10.1371/journal.pone.0323780)
Supplement: S3 File — (DOCX) [file pone.0323780.s003.docx]

**Survey on the impact of public reporting on hospital choice**

| Hello  This questionnaire is intended to investigate the factors that influence hospital choice after the onset of symptoms of acute disease and chronic disease. There are a total of 17 survey questions, and the estimated time required for response is within 10 minutes. Surveys are anonymous and will never be used for any purpose other than research. Thank you for taking your time to complete the survey. |
| --- |

| The followings are questions about choosing a hospital when symptoms of acute myocardial infarction (AMI)  occur. | | | | |
| --- | --- | --- | --- | --- |
| The Health Insurance Review and Assessment Service (HIRA), which is affiliated institution of the Ministry of Health and Welfare, conducts hospital care quality assessments for 36 diseases such as acute diseases (acute myocardial infarction, etc.) and chronic diseases (stomach cancer) and discloses the results. | | | | |
| - **How well are you aware of Public reporting of the HIRA?** - Very well - Some - Little - Not at all | | | | |
| - **Which of the following hospital will you visit if you or your family have AMI?** - Usual hospitals - Nearby hospitals (General Hospital) - Famous hospitals recommended by acquaintances - Hospitals that received good rating for AMI treatment by the HIRA - No idea | | | | |
| - **How will you rate your hospital choice for the following hospitals?** (You are not allowed to give the same score to each hospital. Ex) 4 <- high score) | Low <-> High | | | |
|  | 1 | 2 | 3 | 4 |
| - Usual hospitals |  |  |  |  |
| - Nearby hospitals (General Hospital) |  |  |  |  |
| - Famous hospitals recommended by acquaintances |  |  |  |  |
| - Hospitals that received good rating for AMI treatment by the HIRA |  |  |  |  |
| Since AMI requires immediate treatment, hospital care quality of AMI treatment is evaluated whether the treatment (oral medication, stents, etc.) was done in a reasonable time. The results of the public reporting are classified into five grades according to the score. Smaller numbers indicate better hospitals for the AMI treatment. | | | | |
| - **To what extent would you consider hospital rating for your future hospital choice for AMI treatment?** - Considerably - Some - Not at all | | | | |
| AMI is a life-threatening condition that occurs when blood flow decreases or stops to a part of the heart, causing damage to the heart muscle. The most common symptom is chest pain or discomfort which may travel into the shoulder, arm, back, neck, or jaw. AMI is high-mortality disease and requires immediate treatment. | | | | |
| - **Which of the following hospital will you visit if you or your family have AMI?** - Usual hospitals - Nearby hospitals (General Hospital) - Famous hospitals recommended by acquaintances - Hospitals that received good rating for AMI treatment by the HIRA - No idea | | | | |
| - **How will you rate your hospital choice for the following hospitals?** (You are not allowed to give the same score to each hospital) | 1 | 2 | 3 | 4 |
| - Usual hospitals |  |  |  |  |
| - Nearby hospitals (General Hospital) |  |  |  |  |
| - Famous hospitals recommended by acquaintances |  |  |  |  |
| - Hospitals that received good rating for AMI treatment by the HIRA |  |  |  |  |
| The followings are questions about choosing a hospital in the case of cancer. | | | | |
| - **Which of the following hospitals will you visit if you or your family have cancer?** - Usual hospitals - Nearby hospitals (General Hospital) - Famous hospitals recommended by acquaintances - Hospitals that received good rating for AMI treatment by the HIRA - No idea | | | | |
| - **How will you rate your hospital choice for the following hospitals?** (You are not allowed to give the same score to each hospital) | 1 | 2 | 3 | 4 |
| - Usual hospitals |  |  |  |  |
| - Nearby hospitals (General Hospital) |  |  |  |  |
| - Famous hospitals recommended by acquaintances |  |  |  |  |
| - Hospitals that received good rating for AMI treatment by the HIRA |  |  |  |  |
| Since accurate diagnosis and treatment are important for the patients with cancer, hospital care quality of cancer treatment evaluate whether a biopsy appropriate to the characteristics of each cancer and administration of anticancer drugs have been properly performed. The results of the public reporting are classified into five grades according to the score. | | | | |
| - **To what extent would you consider hospital rating for your future hospital choice for cancer treatment?** - Considerably - Some - Not at all | | | | |
| - **Which of following factors will you consider the most for choosing hospital for cancer treatment?** - Reasonable price - Hospitals received good rates for cancer or cancer treatment from the HIRA - Recommendation of acquaintances - Close distance and convenient transportation - Patients’ satisfaction with hospital services - The size of hospitals and high-tech facilities - Other | | | | |
| The followings are questions about your level of knowledge about AMI. | | | | |
| - **How well are you aware of the symptoms, coping and treatment methods for AMI** - Very well - Some - Little - Not at all | | | | |
| - **What is your information resource of AMI?** - Health professionals - Acquaintances - Mass media such as TV - Internet or books - Other | | | | |
| - **What is the most useful information to cope with AMI (multi-selectable)?** - Preventive measure of AMI - Early symptoms and self-diagnosis of AMI - Hospital information for AMI treatment - Hospital ratings for AMI care quality - Other ( ) | | | | |
| - **Which of following factors will you consider the most for choosing hospital for AMI treatment?** - Reasonable price - Hospitals received good rates for AMI or AMI treatment from the HIRA - Recommendation of acquaintances - Close distance and convenient transportation - Patients’ satisfaction with hospital services - The size of hospitals and high-tech facilities - Other ( ) | | | | |
| The followings are questions to examine the characteristics of survey respondents. | | | | |
| - **When is your year of birth? (Please enter 4 digits of your year of birth.** - **Where are you living?** - Seoul Capital City - Sejong City - Gwangju Metropolitan City - Daegu Metropolitan City - Daejeon Metropolitan City - Busan Metropolitan City Busan City - Ulsan Metropolitan City - Incheon Metropolitan City - Gangwon-do - Gyeonggi-do - Gyeongsangnam-do - Gyeongsangbuk-do - Jeollanam-do - Jeollabuk-do - Chungcheongnam-do - Chungcheongbuk-do - Jeju-do - **What is your gender?** - Male - Female - **What is your highest level of education?** - No learning - Graduated from elementary school (elementary school) - Graduated from middle school - High school graduation - Graduate from university - Graduate graduation or higher - **Are your (or your family member) job health or medical related?** - Yes => Go to Question Job - No => Exit - **Which of the following is your job?** - Doctor - Nurse - Hospital staff (except doctors and nurses) - Employees of public health organizations - Health-related association staff - Other | | | | |
